# A multi-component intervention to sit less and move more in a contact centre setting: a feasibility study

**DOI:** 10.1186/s12889-019-6615-6

**Published:** 2019-03-12

**Authors:** Abigail S. Morris, Rebecca C. Murphy, Sam O. Shepherd, Genevieve N. Healy, Charlotte L. Edwardson, Lee E. F. Graves

**Affiliations:** 1 Research Institute for Sport and Exercise Sciences, Liverpool John Moores University, Liverpool, UK; 2 School of Public Health, The University of Queensland, Brisbane, Australia; 3 Baker Heart & Diabetes Institute, Melbourne, Victoria Australia; 4 School of Physiotherapy and Exercise Science, Curtin University, Perth, Australia; 5 Diabetes Research Centre, University of Leicester, Leicester, UK; 6 NIHR Leicester Biomedical Research Centre, Leicester, LE5 4PW UK

**Keywords:** Feasibility, Workplace, Intervention, Sedentary behaviour, Physical activity, Standing

## Abstract

**Background:**

Call agents spend ~ 90% of their working day seated, which may negatively impact health, productivity, and wellbeing. This study aimed to explore the acceptability and feasibility of a multi-component workplace intervention targeting increased activity and decreased prolonged sitting in the contact centre setting prior to a full-scale effectiveness trial.

**Methods:**

An 8-week non-randomised pre-post feasibility study was conducted. Using a mixed methods approach, focus groups and interviews were thematically analysed to explore the acceptability and feasibility of key study phases, and provide context to agents’ process evaluation and survey responses. The multi-component intervention, conducted in a single call centre, included height-adjustable workstations, emails, education and training sessions, and support from team leaders and a workplace champion.

**Results:**

Six (of 20) team leaders were recruited, with 17 of 84 call agents *(*78% female, 39.3 ± 11.9 years) completing baseline assessments and 13 completing follow-up. High workload influenced recruitment. Call agents perceived assessments as acceptable, though strategies are needed to enhance fidelity. Education sessions, height-adjustable workstations and emails were perceived as the most effective components; however, height-adjustable hot-desks were not perceived as feasible in this setting.

**Conclusions:**

This study has identified unique, pragmatic considerations for conducting a multi-level, multi-component PA and SB intervention and associated evaluation in highly sedentary call agents in the challenging contact centre setting. The intervention was largely perceived positively, with call agents and team leaders describing numerous perceived positive effects on behavioural, health and work-related outcomes. Findings will be of value to researchers attempting to intervene in contact centres and will be used by the current authors to design a subsequent trial.

**Electronic supplementary material:**

The online version of this article (10.1186/s12889-019-6615-6) contains supplementary material, which is available to authorized users.

## Background

High levels of sedentary behaviour (SB) are associated with risk factors for chronic diseases and all-cause mortality in adults, with associations remaining after accounting for levels of moderate to vigorous intensity physical activity (PA) [1–3]. Therefore, in addition to accruing at least 150 min of moderate or 75 min of vigorous PA weekly [4], adults are recommended to minimise time spent sitting for extended periods [4]. The workplace is an appropriate setting to promote PA and reduce SB, as typically, employed UK adults spend up to two thirds of waking hours at work [5, 6]. Contact centres are a priority sector to target, as call agents have higher levels of obesity compared to customer service and office employees [7] and spend up to 90% of their working day seated [8–10]. Moreover, this sitting is often accrued in prolonged periods > 30 min [10] – a pattern detrimentally associated with musculoskeletal discomfort [11, 12] and fasting blood plasma glucose [13]. Two recent multi-component interventions in desk-based workers observed beneficial changes of 40–45 min/8 h workday^− 1^ in occupational sitting and standing, relative to controls at 12 months [14, 15]. These changes were observed alongside significant and beneficial changes to fasting glucose, cardiometabolic risk [16], job performance, work engagement, presenteeism and psychological factors of quality of life and anxiety [15]. Accordingly, this evidence supports the development and evaluation of workplace SB and PA interventions that aim to improve health and work-related outcomes in the 4% (~ 766, 000 adults) of the UK adult population who work in contact centres [17].

Factors contributing to low PA and high SB at work among call agents are multifaceted and include high productivity requirements, sedentary working cultures and sitting-based workstations [18–20]. In contrast to other sectors of desk-based workers (i.e. non contact-centre), however, call agents are less able to sporadically break up their sitting time and move at work due to a physical connection to their computer via headsets, a lack of autonomy over their workload, and/or the need to maintain high call volumes to meet continuously monitored productivity targets [18]. It is important therefore that the development of interventions to reduce prolonged sitting in this sector take into account these multi-level and interacting influences on behaviour [21].

While multi-component interventions have successfully reduced occupational sitting time in desk-based workers [14, 15], limited research has investigated the effect of PA and SB interventions in contact centre call agents [18]. The provision of height-adjustable workstations reduced call agents’ self-reported occupational sitting time [8] and increased objectively-assessed productivity [22] compared to seated workstation controls over 6 months. Similarly, a multi-component pilot study in 16 call agents, which also included the provision of height-adjustable workstations, observed favourable changes in call agents’ self-reported workplace sitting and standing time compared to 15 seated controls after 1, 4 and 19 weeks [23]. These findings are however based on small samples and subjective measures of PA and SB. There is a need for more robust evaluation of PA and SB interventions in contact centres.

Development and piloting is recommended prior to the definitive evaluation of complex interventions [24, 25]. In line with the aims of delivering a pilot and feasibility trial [26], the present study focused on exploring the acceptability and feasibility of recruitment, data collection, and the intervention components and delivery, therefore, effectiveness data is not presented [27]. Such systematic development allows researchers to experience the delivery of a small-scale version of the intended subsequent trial [28] and seeks to enhance the likely effectiveness and sustainability of the trial [29, 30]. To date, no PA or SB intervention in the contact centre setting has been developed in this manner [8, 22, 23].

Following original formative research by the present authors [18], this study aimed to explore the acceptability and feasibility of delivering and evaluating a multi-component SB and PA workplace intervention in the contact centre setting prior to a full-scale effectiveness trial. Objectives were to assess response, recruitment and attrition rates, completion rates for all outcome measures, and the acceptability and feasibility of the intervention from participant and organisational perspectives [28, 29]. The findings will be used to justify and refine the design and delivery of a larger trial understanding the impact of a multi-component SB and PA workplace intervention on changes in behaviour and health, wellbeing, and productivity indicators.

## Methods

### Study design

Data for this 8-week non-randomised pre-post feasibility study was collected between July–September 2017. The study is reported in line with the Template for Intervention Description and Replication (TIDieR) checklist to enhance transparency and replicability for future trials [31]. Liverpool John Moores University (17/SPS/003) granted ethical approval.

### Recruitment

**Fig. 1:**
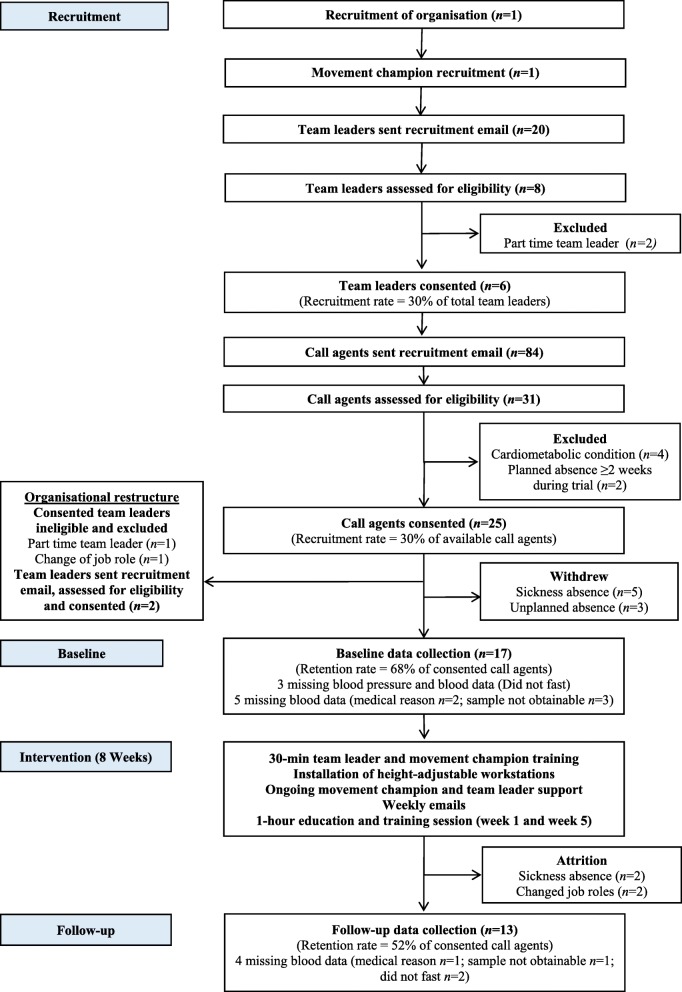
CONSORT flowchart of study recruitment, retention and assessment data collection

Recruitment was required for the organisation, a movement champion, team leaders, and individual call agents (see Fig. 1).

### Recruitment of organisation

A contact centre (> 500 employees) who contributed to formative research [18] expressed interest through informal discussions. The research team discussed the study aims, objectives, requirements and feasibility considerations with a gatekeeper from the organisation, who consented to onsite recruitment, data collection and intervention delivery during work hours. The gatekeeper identified a member of middle management for the role of centre contact to the research team and participants, who agreed to support recruitment, data collection and intervention delivery. The gatekeeper approved the centre contact to organise offline time for agents to engage in data collection and relevant intervention components. One office floor in the contact centre dedicated to inbound call agents was identified. Across the office floor were 20 work pods, each housing 14 call agents, with one team leader per pod. Accordingly, the floor housed 20 team leaders and 280 call agents.

### Recruitment of movement champion

A movement champion was appointed to provide daily verbal support for agents to sit less and move more, and encourage team leaders to promote the sit less and move more message to their agents. The gatekeeper and centre contact identified a staff member in the organisation to be approached for the role. The staff member agreed and met the inclusion criteria: a) full time staff member in a support role in the organisation (≥0.8 full time or part time equivalent worker), b) access to a work telephone and desktop computer with internet, c) aged ≥18 years, d) ambulatory, e) no planned absence for ≥2 weeks during the intervention, f) not pregnant, and g) provided written informed consent for the role.

### Recruitment of team leaders

In May 2017, on behalf of the research team, the centre contact emailed the 20 team leaders a participant information sheet and invitation to a researcher-led, drop-in session that provided an overview of the study and intervention. Team leaders were informed that their call agents would only be invited to participate, if they, the team leader, were interested and eligible to participate. Team leaders had one week to express interest in participating to the centre contact by email, telephone or an expression of interest form, with two email reminders sent during this period.

### Recruitment of call agents

In May–June 2017, on behalf of the research team, the centre contact emailed call agents managed by an interested and eligible team leader. The email included a participant information sheet and invitation to two researcher-led, drop-in sessions that provided an overview of the study and intervention. Call agents had two weeks to express interest in participating to the centre contact by email, telephone or an expression of interest form, with two email reminders sent during this period.

### Eligibility and selection

The research team screened interested team leaders and call agents face-to-face or by telephone for the following eligibility criteria: a) full time staff member (≥0.8 full time or part time equivalent worker) in a team leader or call agent role, respectively, b) access to a work telephone and desktop computer with internet, c) aged ≥18 years, d) ambulatory, e) no planned absence for ≥2 weeks during the intervention, f) not pregnant, g) no known cardiovascular or metabolic disease (agents only). Interested employees were notified of study acceptance via an email from the centre contact on behalf of the research team. Written informed consent was obtained and baseline assessment scheduled. Participants were allocated a unique identification number for assessments including focus group contributions. There was no racial or gender bias in participant selection.

### Intervention

#### Theoretical basis and intervention development

In line with the socio-ecological model [21, 32], factors influencing call agents’ workplace PA and SB, identified in part by formative research [18], were targeted via intervention components at the organisational, environmental, interpersonal and intrapersonal level [21] (Table 1). Factors were mapped to pragmatic intervention components within the behaviour change wheel to enhance agents capability, opportunity and motivation to sit less and move more at work [33], and progress towards accumulating 2–4 h/day of standing and light activity (light walking) during working hours [34].

**Table 1:** Intervention components and delivery timeline

Intervention component	Level	0	1	2	3	4	5	6	7	8
Education and training session for team leaders and the movement champion	Intrapersonal/ Interpersonal/ Organisational	**x**								
Health check feedback	Intrapersonal		**x**							**x**
Education and training session for call agents	Intrapersonal		**x**				**x**			
Emails	Intrapersonal		**x**	**x**	**x**	**x**	**x**	**x**	**x**	**x**
Height-adjustable workstations	Environmental		●	●	●	●	●	●	●	●
Team leader support	Interpersonal		●	●	●	●	●	●	●	●
Movement champion	Interpersonal		●	●	●	●	●	●	●	●

0 to 8 represents the week number. Week 0 indicates post-baseline but pre-intervention delivery

**x** Administered intervention component ● Ongoing intervention component

### Intervention procedures

#### Organisational level

To demonstrate organisational buy-in and foster a supportive environment, team leaders and call agents were told at recruitment that senior management had approved the appointment of a centre contact and a movement champion, the installation of height-adjustable workstations, and offline time for agents to engage in data collection and relevant intervention components.

### Environmental level

#### Installation of height-adjustable workstations

Following baseline, the research team installed 14 height-adjustable workstations (Posturite, DeskRite 100 small, UK) during work hours. Call agents had a height-adjustable workstation installed onto their desk if they had an occupational health need (determined by a prior display screen assessment [35]) or a technical need (i.e. hardware or software requirement) that would prevent them from moving between their desk and a hot-desk on their pod that had a height-adjustable workstation installed on it. Participants without an occupational health or technical need only had access to a height-adjustable workstation installed onto a hot-desk in their pod. The feasibility of this hot-desk system was explored during process evaluation, as a hot-desk policy was not in place at the company. The computer monitor(s) and keyboard were housed on the workstation, which could be quickly raised and lowered by hand to enable seated or standing work. Participants were not prescribed an amount of time to use the workstation. Each workstation had a laminated sheet attached to its surface detailing the intervention aim to sit less and move more, and safe ergonomic postures during seated and standing use, as recommended [36]. After follow up data collection, the research team uninstalled the workstations.

### Interpersonal level

#### Team leader and movement champion support

Between baseline and height-adjustable workstation installation, team leaders and the movement champion were invited to a 30-min researcher-led, education and training session. The session reinforced the intervention aim for call agents to sit less and move more at work in accordance with workplace recommendations [34], provided a rationale for the intervention, and an overview of the intervention timeline. Team leaders and the movement champion were engaged in guided discussions regarding their respective roles. Team leaders were specifically educated, trained and encouraged to a) encourage walking in their one-to-one and team meetings with agents, b) discuss agent experiences of the intervention during one-to-one and team meetings, c) provide daily verbal support and encouragement to agents to sit less and move more, and d) forward a weekly intervention email to their agents. The movement champion was specifically encouraged to provide daily verbal support for agents to sit less and move more, and encourage team leaders to complete the above actions. Team leaders and the movement champion left the session with a laminated information sheet that detailed the intervention aim, timeline and components, and suggested strategies to promote their agents to sit less and move more at work.

#### Weekly emails

Team leaders forwarded weekly intervention emails to their participating call agents. The emails, which contained a non-modifiable infographic, were designed by the research team and emailed to team leaders via the centre contact. The infographic encouraged and suggested ways for call agents to break up prolonged periods of sitting and be active during scheduled breaks and lunch. Suggestions included breaking their sitting time after each phone call, using the height-adjustable workstation, and walking breaks. Team leaders were instructed to copy the research team into the emails to assess fidelity.

### Intrapersonal level

#### Education and training sessions

The centre contact, on behalf of the research team, emailed the call agents, movement champion and team leaders (for information only) a calendar invite to a 40-min researcher-led, group education and training session in intervention week 1 and 5. Sessions reinforced the intervention aim to sit less and move more at work in accordance with workplace recommendations [34]. Sessions introduced (week 1) and reinforced (week 5) the benefits of moving more and sitting less each day at work and the risks of prolonged sitting and standing. Using the intervention components as a point of departure, agents engaged in guided discussions to identify how they could utilise each intervention component to facilitate their behaviour change. Agents were given the opportunity to discuss their intervention experiences, including barriers to sitting less and moving more. In week 1 agents wrote a short-term goal to help them sit less and move more at work, for example, ‘*I will go for a walk during my lunch break tomorrow’*. This goal was discussed and reflected on in the week 5 session.

### Data collection

Each call agent attended a 1-h assessment in a designated room at work at baseline and 8 weeks (follow-up). For convenience and to promote arriving in a fasted state, agents were allocated an arrival time between 08:00–12:00 on Monday, Tuesday or Wednesday, with the time and date replicated at follow up. To promote privacy, confidentiality and comfort, screens were used and trained researchers conducted all assessments. This 1-h session included cardiometabolic health and anthropometric assessments, survey completion, and fitting each agent with an activPAL monitor (PAL Technologies, Glasgow, UK), to continuously assess PA and SB for 7 days. Prior to data collection, agents were instructed via email to wear light clothing, fast for 10 h, avoid the consumption of alcohol, tea and coffee for 12 h, and avoid strenuous exercise for 24 h. At baseline, the email included a food and fluid form for agents to complete across the 24 h prior to their assessment. The form was collected by the research team and returned to the participant before follow up, with instructions to replicate their food and fluid intake across the 24 h prior to the assessment.

### Outcomes

#### Recruitment, retention and attrition

Agents’ intervention pathway and completion rates for all outcome measures were assessed.

#### Acceptability and feasibility - focus groups and interview

Participants were invited to a focus group (call agents, team leaders) or interview (movement champion) within 2 weeks of the follow up assessments to assess acceptability and feasibility of the recruitment strategy, data collection procedures and intervention components. The focus groups and interview were conducted in homogenous occupational groups to promote open discussions, to elicit in-depth insights into participant perspectives and experiences, and to provide context to agents’ acceptability and feasibility survey responses [37]. Team leaders and the movement champion also reflected on barriers or facilitators experienced in implementing their respective roles. The protocol for delivery was standardised by using a semi-structured focus group/interview schedule to maintain a level of commonality across the groups [38], while allowing flexibility in the order and sequence of questions to promote participants to respond openly and freely, using probes where appropriate to elicit depth from responses [39]. Four focus groups were conducted with call agents, two with team leaders, and one interview with the movement champion, with each audio recorded, transcribed verbatim and anonymised during this process.

#### Acceptability and feasibility - surveys

At follow up, call agents completed a 33-item questionnaire, containing 5-point Likert-type questions adapted from a previous trial [40]. Response scales ranged from [1] strongly agree to [5] strongly disagree. To help establish suitable procedures for delivering the intervention in future trials and to build on the qualitative data, survey items explored the acceptability and feasibility of data collection and each intervention component, and agents’ willingness to receive each intervention component in the future. The assessment of the perceived effectiveness of each intervention component was viewed as an acceptability index, based on previous positive associations observed between perceived effectiveness and actual effectiveness [41].

#### Anthropometry: Stature, body mass and body composition

Using standard anthropometric techniques [42] and with call agents wearing light clothing and no shoes, stature was measured to the nearest 0 .1cm using a portable stadiometer (Marsden HM 250P, Leicester Height Measure, Seca Ltd., Birmingham, UK) and body mass to the nearest 0.1 kg using a calibrated mechanical flat scale (Seca Clara 803, Seca Ltd., Birmingham, UK). Body mass index was calculated as mass divided by stature (kg/m^2^). Waist and hip circumference were measured to the nearest 0.1 cm using an inelastic anthropometric tape (Lufkin W606 PM, Apex Tool Group Ltd., Sparks, MD, USA). For all outcomes, if the difference between the two measures taken exceeded > 1%, a third measure was taken and the mean calculated.

#### Cardiometabolic markers

In accordance with standardised guidelines [43] and after 15 min of seated rest, an automated sphygmomanometer (Omron, Omron Healthcare, UK) measured resting blood pressure on the brachial artery of the bare right arm two times, at one minute intervals. If the difference between the two measures was ≥5 mmHg, a third measure was taken and the mean calculated. A 15 ml fasting blood sample was taken from the antecubital vein of one arm using standard venepuncture technique (Vacutainers Systems, Becton-Dickinson, USA). Samples were collected into vacutainers containing edetate disociom or lithium heparin, immediately labelled with the unique participant number, and stored on ice during transportation to University laboratories for later analysis of glucose, total cholesterol and triglycerides.

#### Survey measures and outcomes

Call agents completed a non-validated survey adapted from a previous trial [44] to assess sociodemographic (age, gender, ethnicity, marital status, education), work history (employment history, employment status, job category, hours worked, main work tasks) and work environment (number of people in their office) characteristics. In addition, agents self-reported presenteeism using the Work Limitations Questionnaire [45], absenteeism using the Health and Work Questionnaire [46], job satisfaction using a general job satisfaction tool [47], musculoskeletal symptoms during the last 7-days, three and twelve months, across nine symptom sites, using the 27-item Nordic Musculoskeletal Questionnaire [48, 49], remembered and experienced wellbeing using the Pemberton Happiness Index [50], and, health and quality of life using the EQ-5D questionnaire [51].

### Behavioural outcomes

#### Sitting, standing and moving time

Call agent’s work and leisure time sitting, standing and walking, plus sit-to-stand transitions, time accrued in sitting bouts ≥30 min and steps taken were assessed continuously for 7 days using an activPAL monitor. Placement was standardised to the anterior midline of the upper right thigh, with monitors inserted into a flexible waterproof sleeve (PAL Technologies, Glasgow, UK) and attached using a hypoallergenic waterproof adhesive strip (Tegaderm 3 M, Bracknell, UK). Agents were provided additional waterproof sleeves, adhesive strips and an instruction leaflet on correct placement should they wish to change the dressing. To promote wear compliance and derive work times, agents were instructed to report the time they started and finished work (when applicable), went to bed, went to sleep, woke up and got out of bed in a daily diary [52]. Agents were instructed to return their monitors and completed diaries to the centre contact at the end of the monitoring period.

### Analyses

#### Acceptability and feasibility

Taking a phenomenological approach [53] and in accordance with the study aim, deductive thematic analysis explored patterns and identified themes within the raw focus group and interview data, in relation to participant perceptions of the acceptability and feasibility of the recruitment strategy, data collection procedures and intervention components [54]. Exploration of multiple stakeholder perspectives provides broader insights than a single stakeholder group, and perspectives can be contextualised in relation to the wider social and environmental context [53]. During familiarisation, transcripts were read, initially coded and further analysed to identify higher-order themes using NVivo version 11 (QSR International Pty Ltd). Sub-themes emerged through an inductive process when transcripts were re-read to add rich context to the research question beyond the pre-defined categories [54]. Triangulation meetings between authors (AM, LG, RM) discussed emerging themes and refined the thematic framework, with this process enhancing the credibility of the analysis process [55]. Findings are reported in line with the consolidated criteria for reporting qualitative research (COREQ) checklist [56]. Process evaluation surveys were analysed using SPSS version 22 (IBM, New York, USA) to describe the frequency (%) of distribution across responses [57]. Baseline sociodemographic and work characteristics, and anthropometric, cardiometabolic, blood pressure, activPAL and survey data were analysed to describe the sample. Completion rates of all outcome measures at baseline and follow-up were identified to inform the acceptability and feasibility of the data collection procedures.

### Behavioural outcomes

Activity data was downloaded using manufacturer software (PAL technologies, Glasgow, UK) and processed using ProcessingPAL-V1.0, Leicester, UK. This software using a validated algorithm to separate valid waking wear data from everything else (i.e. time in bed, prolonged non-wear, invalid data). A day was considered invalid if there was limited postural variation (i.e. ≥95% of wear time in one activity), limited steps (< 500 steps/day) or < 10 h valid waking wear time [58]. This algorithm has demonstrated almost perfect (k > 0.8 for 88% of participants) agreement with the traditional diary method [58]. Summary data from the algorithm was quality checked using heat maps against participant diaries to check whether the algorithm had successfully been applied to the data [52]. Corrections were made if the self-reported waking time was not consistent with the algorithm output [58]. Participants’ workdays and times were manually entered into a csv template and uploaded into the software, which enabled the calculation of work time PA and SB.

## Results

Acceptability and feasibility results from the surveys and focus groups/interview are presented together, with verbatim quotes attributed by job role (AG = Agent P1–16, TL = Team Leader P1–5, MC = Movement Champion) and data collection method (FG = Focus group, I=Interview). Mean interview and focus group length was 37.1 ± 7.4 min.

### Recruitment and retention

Of the 20 team leaders who received the recruitment email, 8 expressed interest (40%) with 6 eligible (30%: Fig. 1). Subsequently, of the 84 call agents who received the recruitment email, 31 expressed interest (37%) with 25 eligible (30%).

### Recruitment – Team leaders

Recruitment occurred at a time of high workload, which resulted in low team leader attendance at the researcher-led, study information session (4 of 20 = 20%).

*“I think I was so busy when* [recruitment] *first came round.”* (TL4 FG).

*“…As an organisation in the last 6 months we’ve gone through a real change in workload, so our workload has been quite heavy.”* (TL2 FG)

Consequently, low team leader engagement during recruitment appeared to negatively influence team leader perceptions of the burden of the intervention.

*“I think a lot of people would have looked at it* [recruitment email] *and thought more work if I* [am] *being honest with you.”* (TL3 FG)

To promote team leader recruitment, one team leader suggested establishing a clear overview of the organisational structure and engaging additional stakeholders, such as team leader managers.

*“So I think if that* [information session] *had been delivered to our* [team leader managers], *then to the team managers within the* [manager] *meetings* […] *You’d probably get more backing from everybody because we’re all kind of,* […] *one person will say ‘oh I’ll do it’ ‘oh well I’ll do it’ and then everybody decides that they’re going to do it.”* (TL1 FG)

### Recruitment - call agents

All agents reported a high volume of daily work emails and perceived the lengthy recruitment email as ineffective.

*“We do get a lot of emails* […] *we get a lot of junk emails as well, because people send emails out saying they’re doing* […] *all sorts of rubbish, and you just think like, literally, I just need to get on with my work.”* (AG16 FG)

To promote call agent recruitment, clear, concise and engaging recruitment materials and face-to-face interaction were suggested.

*“I think personally, you should just come in to team meetings and explain what you are, what you’re after, and then sign people up there and then*.” (AG20 FG)

One team leader described that many agents felt deterred from expressing interest or were unable to participate due to the eligibility criteria requiring the absence of cardiovascular or metabolic disease.

*“…other people wanted to do it* [the study] *but obviously they didn’t meet the criteria,* […] *I think it would have been really great if some of the others, but obviously because of the medical reasons they couldn’t be involved in it, but it would be really great moving forward if we could kind of encourage that* [participation].” (TL2 FG)

Further, the two researcher-led, drop-in sessions occurred during ‘red alert’ where call volumes in the centre are unexpectedly high, and non-essential offline time is prohibited. Consequently, as offline time to attend the sessions was considered non-essential by the organisation, the agents were prevented from attending.

*“We’ve been so busy lately on the phone that even our own normal team leader meetings we’ve not been able to get offline for.”* (AG10 FG)

### Data collection

Of the 25 consenting agents, 17 (68%) and 13 (52%) completed baseline and follow-up, respectively, with attrition due to sickness, unplanned absence and job role changes (Fig. 1). Call agents reported the survey completion as feasible (Additional file 1 Table S1) with no missing data from those issued surveys (17/17 at baseline, 13/13 at follow-up). Anthropometric assessments were reported as feasible (Additional file 1 Table S1), though one agent felt uncomfortable when a member of the opposite sex took their measurements.

*“I felt a bit uncomfortable having, it was a guy doing my measurements, and I felt a bit uncomfortable with that* […] *I would have preferred a woman to do that, but maybe again, that’s just me* […] *just because I’m self-conscious about the way I...Because I know I’m overweight anyway, so I just felt a bit, you know. It made me more uncomfortable.” (*AG18 FG)

Despite most agents reporting the blood pressure assessment, blood sampling and associated fasting as feasible (Additional file 1 Table S1), medical factors and forgetting to fast led to missing data (Fig. 1). To promote compliance to fasting, agents suggested a text message reminder 24 h before each assessment.

*“On the first* [assessment]*, I didn’t fast* […] *I think a text would be really good, because* [you forget] *if you’re off for a couple of days.”* (AG23 FG)

Most agents reported the 7-day activPAL monitoring as feasible (Additional file 1 Table S1). Fifteen of 17 agents (82%) and 10 of 13 agents (77%) fitted with an activPAL at baseline and follow up, respectively, provided ≥3 valid days of data (Table 2). Ten agents provided ≥3 valid days of data at both time points and 17 agents provided ≥1 valid workday at baseline. Call agents were predominantly female, White British, full-time employees, educated to tertiary level with ≥3 year tenure (Table 2). At baseline, on average, agents were pre-hypertensive [43], overweight [59], had an elevated waist circumference [60], were sedentary for > 10 h per day and spent 82% of work hours sitting, 15% standing and 3% stepping (Table 2).

**Table 2:** Baseline characteristics of participating call agents (*n* = 17)

Female	14 (78)
Age (years)	39.3 ± 11.9
White British	15 (83)
Married	7 (41)
Full-time employee	16 (94)
Tenure in current role ≥3 years	10 (56)
Tertiary education	11 (61)
Daily hours worked (h/day)	7.4 ± 1.0
Weekly hours worked (h/week)	37.3 ± 2.1
Systolic blood pressure (mmHg)	124.5 ± 12.9
Diastolic blood pressure (mmHg)	86.6 ± 7.2
Body mass index (kg/m^2^)	33.6 ± 8.3
Waist circumference (cm)	111.4 ± 32.4
Hip circumference (cm)	120.5 ± 19.3
Waist-to-hip ratio	0.92 ± 0.25
Activity outcomes Daily	
Waking wear time (min/day)	906.6 ± 80.3
Valid wear (days)	5.0± 1.8
Sitting time (min/day)	642.0 ± 88.2
Standing time (min/day)	178.2 ± 76.8
Stepping time (min/day)	86.4 ± 39.6
Steps (steps/day)	7215 ± 3507
Sit-to-upright transitions/day	56.1 ± 19.1
Time sitting in bouts < 30 min (min/day)	306.0 ± 96.6
Time sitting in bouts ≥30 min (min/day)	336.0 ± 154.8
Workplace	
Total work time (min/day)	473.9 ± 73.9
Valid wear (days)	3.1 ± 1.3
Sitting time (min/day)	376.1 ± 136.3
Standing time (min/day)	72.4 ± 23.3
Stepping time (min/day)	25.4 ± 13.1

Data is presented as n (%) or mean ± SD

### Intervention components and delivery

#### Organisational level

Team leaders were positive about the appointment of a centre contact who managed the scheduling of agents’ study-related offline time.

*“From a manager perspective it was good that the exceptions* [for offline time] *were put in by centre contact, rather than us trying to call those in.”* (TL3 FG)

Despite being told the study had organisational support, some agents’ desire to sit less and move more at work appeared influenced by their awareness of meeting productivity targets.

*“You're literally doing calls for eight hours, you're very restricted with the time that you have, because whatever you're signed into on the PC is a statistic that goes towards your end-of-month, and if you're not where you're supposed to be, it doesn't go in your favour, to be honest.”* (AG16 FG)

Lastly, participants indicated that to receive a workstation modification (e.g. ergonomic chair), current organisational processes required agents to have a display screen equipment assessment [35] and existing musculoskeletal or chronic health problem. With respect to this, all stakeholders believed that implementing height-adjustable workstations as a preventative measure would demonstrate increased organisational buy-in and may mutually benefit agent health and the business.

*“The obvious one there is the price of these desks* [height-adjustable workstations] *ever coming onto site, what do we have to do? We’ve spent between £900 and £3,000 on a chair that is adapted for that individual person, so special chairs from a workstation assessment like back problems, it’s like right workstation assessment, you’re recommended to have this chair, some of them are absolutely fantastic all singing, all dancing, they do everything apart from answer the phone call for you… compared to £170* [height-adjustable workstation cost] *that could do the same thing.”* (TL3 FG)

### Environmental level

#### Initiation, maintenance and termination of height-adjustable workstations

The majority of call agents reported the height-adjustable workstations as somewhat-to-very effective for helping them to sit less at work (Table 3), easy to use, and most felt comfortable using the workstation in the presence of others (Additional file 1 Table S1). Seeing other agents use the workstation in the standing position was the most common trigger for standing work, and this appeared more prominent among teams with multiple height-adjustable workstation users.

*“We kind of prompted each other as well, don’t we? Because when one went up, you noticed the other one went as well.”* (AG14 FG)

*“AG5, used* [the height-adjustable workstation] *a lot. They would stand up a lot, and I think with us, it was definitely more support because more of us had them.”* (AG18 FG)

**Table 3:** Participating call agents’ perceived effectiveness of each intervention component

	1	2	3	4	5
How effective did you find the height adjustable workstation in helping you to sit less and move more at work?	73%	9%	–	–	18%
How effective did you find the movement champion in helping you to sit less and move more at work?	36%	27%	27%	9%	–
How effective did you find the weekly team leader emails in helping you to sit less and move more at work?	64%	27%	–	9%	–
How effective did you find the weekly team meeting in helping you to sit less and move more at work?	36%	9%	18%	9%	18%
How effective did you find the walking 1:1 meetings with your team leader in helping you to sit less and move more at work?	9%	18%	27%	–	36%
How effective did you find the two education and training sessions in helping you to sit less and move more at work?	91%	9%	–	–	–

1 = very effective, 2 = somewhat effective, 3 = neutral, 4 = somewhat ineffective, 5 = very ineffective

In contrast, during focus groups, several agents reported feeling self-conscious during standing work among seated colleagues, which appeared to negate workstation use over time. This perception of social conformity to seated work was largely attributed to low participant numbers within teams.

*“Maybe that’s why, because no one else was doing it* [standing], *and you just feel a bit, a little bit daft just standing up.”* (AG23 FG)

Accordingly, a common challenge described by agents was keeping motivated to use the workstation in the standing position. Compounded by the lack of social support, some agents forgot to use their workstation in the standing position and reverted to seated working habits over time.

*“*[Initially] *I was like using it quite a lot. As the sort of eight weeks went on, I slowly and slowly used it less and less, or I would forget to use it. Like I’d get to like six o’clock in the evening, and I’d be like, “I’ve not even stood up today”. I’d be like, Right, let’s stand up.”* (AG16 FG)

In contrast, several agents described having a daily routine across the intervention of frequent postural changes between sitting and standing, primarily triggered by work-based cues including times of the day and dealing with challenging customer calls.

*“I soon got in a routine where I knew I was coming in and I was eating breakfast, maybe half hour or an hour, get up, and then that would be me up* [standing] *pretty much the majority of the day, sit down after my lunch and then back up again. I just fell into that routine.”* (AG8 FG)

*“I find that if you’ve got a really shouty customer or anything like that, you’ve got an awkward account and you need to assert yourself, it* [the height-adjustable workstation] *went straight up.”* (AG14 FG)

#### Hot-desk feasibility

Call agents with a height-adjustable workstation installed onto their desk (*n* = 10) believed that ownership of an individual workstation was important for enhancing acceptability and feasibility of the workstations. Two of the four call agents who only had access to a height-adjustable workstation on a hot-desk on their pod indicated that they did not use the workstation at all during the trial, and reported the height-adjustable workstation as very ineffective (Table 3). The main barrier influencing hot-desk use for these agents was the time to move equipment and belongings between desks. One team leader described how switching between desks could negatively affect agent productivity, due to the specialist equipment and software required to conduct their job efficiently.

*“People get used to their own comforts and they make their own kind of their desks, they arrange their desks how they need it so it goes with their flow and it can really, really, it can be quite a big upheaval for somebody to move their workstations* […] *they’ve got their own equipment like mouse mats or something like that then it can take some time for them to set up that workstation how they need it, you’re losing time.”* (TL1 FG)

#### Perceived effects of height-adjustable workstations

While a minority of agents reported that they had more musculoskeletal symptoms on the days they used the workstations (Additional file 1 Table S1), many agents described that standing work contributed to perceived reductions in musculoskeletal symptoms. Most agents were willing to continue to have access to the height-adjustable workstations, all agents would have a workstation if offered by their employer, and, all agents were willing to receive further advice and guidance for using the workstation to optimise health (Additional file 1 Table S1).

“*I used to always finish my shift, and I’d have a pain right down the middle of my back, that I haven’t got that when I’ve been using the desk* [height-adjustable workstation]*. So on them five days when I wasn’t able to stand, the pain was back, but then when I was able to use the desk again, it’s gone.”* (AG20 FG)

A minority of agents felt more tired on the days they used the height-adjustable workstations (Additional file 1 Table S1), though other agents perceived that workstation use reduced their levels of fatigue across the working day, which was consistent with team leader’s perceptions.

*“So you get a lull in the day don’t you when you're tired* […] *I've noticed because P9, one of my guys I can see when, if we have a pocket of availability and he's on an early shift by, after his lunch* […] *I need to get him a call through because I can see* [he’s tired]*, but I don’t see that now because he stands up.*” (TL1 FG)

Agents strongly disagreed that use of the workstation had a detrimental impact on their work-related productivity or work quality (Additional file 1 Table S1) and there were no participant withdrawals from the intervention due to adverse events. Work-related benefits from using the workstations, perceived by agents and team leaders, included improved projection and tone of voice while standing on calls, which was deemed important as interaction between agents and customer’s is primarily based on verbal communication.

“*It is all vocal, and like they keep saying to us over the years, “Smile on a call, because the customer will hear it”. The same with stand*[ing] *up, you project your voice a bit more when you need to be assertive.”* (AG14 FG)

One team leader identified that their call agent appeared more empowered while standing to deal with challenging calls. This was reflected by several agents who described greater confidence and assertiveness while standing during calls, which they felt benefited their call control.

*“Do you know one thing that I noticed looking back now, when P13 had some of his more difficult conversations the desk* [height-adjustable workstation] *would go up* […] *and he would stand, and I think that gave him a sense of empowerment.”* (TL4 FG)

“[Using the height-adjustable workstations] *you feel more confident. That’s going to help you with an awkward call, and you put your foot down verbally* […] *you’re feeling better, so you’ve probably got more call control.*” (AG14 FG)

Agents and team leaders suggested that improved call control helped performance indicators, with a team leader describing how one agent displayed reduced average handling time across the study.

“[Call agent] *really benefitted from it* [use of the height-adjustable workstation]*. He liked it so much and it helped him, in fact it helped him you know reduce his AHT* [average handling time] *so he did really well, yeah he’s made some big, big reductions.”* (TL5 FG)

### Interpersonal level

#### Weekly emails

From the 8 weekly emails to be sent by the 6 team leaders, the research team received 28 out of 48 (58%). Team leaders perceived the emails as a prompt to talk to their agents about the intervention, and a useful resource to demonstrate their buy-in to the intervention.

*“The only thing that I was doing was when the mails were coming through on a Monday, that’s when I would pick up with P13 so that would be the catalyst for the conversation with P13 to tell him, or ask him how it’s going, that mail was a conversation starter for me to be fair.”* (TL4 FG)

Agents typically found the weekly email easy to digest, aesthetically pleasing and useful for increasing their knowledge and awareness of SB and PA. Accordingly, most agents found the emails somewhat-to-very effective in helping them to sit less and move more (Table 3) and were willing to receive weekly emails in the future (Additional file 1 Table S1).

*“I’ve never, the whole time I’ve been here, sat and done foot exercises or leg exercises under my desk* […] *but it* [the weekly email] *did trigger that often and I have been doing it and I have found it beneficial and I wish I’d done it from the get go you know, it would have been a lot better for me because some days my legs have been that swollen I’ve not been able to barely walk so it’s made a huge big difference.”* (AG10 FG)

### Movement champion support

The movement champion attended the team leader training session and the first agent education session, yet felt it was challenging to consistently implement their role and engage and prompt the agents. This was attributed to the agent’s varied shift patterns, break schedules, and dispersion across the office.

*“…for me it* [the intervention] *was a little bit messy because there were like stragglers and people on different teams* […] *that’s the bit that made it difficult to kind of remember exactly who was on it and who you were prompting.”* (MC I)

Most agents and team leaders felt it was important to have a movement champion, yet, consistent with the movement champion’s perceptions, were often unsure of the movement champion’s role, with one team leader expressing the need to promote greater agent-movement champion interaction.

“*From* [Movement champion’s] *point of view it would be good to make sure that they’re following through and checking on those individuals, say are you sitting are you standing, how’s it going, because I haven’t seen any of that.”* (TL1 FG)

Agents typically reported little-to-no interaction with the movement champion, and agents who did interact with the champion described how the champion’s prompts centred on sitting reduction and workstation use, over promotion of active break times.

*“…if* [the movement champion] *come round to promote movement, and seeing P05 and P18 stood up using them* [the height adjustable workstation] *she wouldn’t have said anything because she sees them using them.*” (AG10 FG)

Agents were willing for the movement champion to continue in their role (Additional file 1 Table S1) but suggested localised champions within teams would increase the perceived effectiveness of this component (Table 3), provide them with greater support, and overcome the challenge the champion faced with engaging agents across shift patterns and office locations.

“*If it’s on your team it’s more relevant,* [Movement champion] *has so much else to do, its finding the time to do it when the people that’s they’re targeting are all there* […] *it’s not always easy.”* (AG11 FG)

### Team leader support

Most agents were willing to receive future team leader support to sit less and move more during team and one-to-one meetings (Additional file 1 Table S1). Despite this, the amount of team leader support appeared inconsistent, and agents identified the weekly team leader meetings and walking one-to-one meetings as the least effective intervention components (Table 3).

*“…walking one-to-ones, that didn’t happen. I really wanted to do one of them.”* (AG20 FG)

*“We had our team meeting, and* [team leader] *was like, “Right, guys, rather than sitting down today, we’re going to go outside”. So we all walked and went to the grassy area outside, and it was a nice day, we had our team meeting out there, and then he made us all do like five star jumps, and it was just a laugh* […] *It was something different* […] *before that, I would literally just get up out of one seat, go to like a break-out room and sit down in another seat, get my phone out, probably just go on my phone for like fifteen minutes or something.*” (AG16 FG)

Agent perceptions appeared consistent with team leaders. While some team leaders reported infrequent intervention-related conversations with agents, others described how they encouraged active team and one-to-one meetings, contributed additional information to the weekly emails, and, provided frequent, ongoing encouragement to use the height-adjustable workstation.

*“For me it was more around meetings, like 1:1 coaching sessions, not necessarily walking them but let’s get up from our desks let’s get up and go somewhere else and it wasn’t always the nearest break out area, it was lets go somewhere that we don’t normally go we got at least a couple of minutes’ walk there and back.”* (TL3 FG)

Two team leaders did not attend the team leader training session, which appeared to affect their knowledge of the intervention and subsequent promotion of the intervention aims to their agents.

“*I think for me personally from the very beginning, I probably would have liked, I know we said about a brief, but I probably would have liked a bit more of a run down as I was very unsure of what it was that I was signing up to for at least 2 or 3 weeks.”* (TL1 FG)

### Intrapersonal level

#### Education and training sessions

Agents perceived the education and training sessions to be very effective for helping them to sit less and move more at work (Table 3). Agents found the sessions motivating, informative and enjoyed the social interaction with other agents, with the majority of agents willing to attend further education and training sessions (Additional file 1 Table S1). Thirteen agents (76%) attended the week 1 session and 10 agents (59%) the week 5 session.

*“I felt really motivated at the end of that* [training session]*. Like I came out, and me and P21 went for a walk, like with our cigs. We decided to go for a walk around the building smoking, rather than waiting there, and for about a week I was doing that on all my lunch, like putting my headphones in and going for a walk.”* (AG23 FG)

Willingness to attend further education and training sessions appeared to be influenced by the incentive of offline time at work, as the majority of agents appeared reluctant to relinquish personal time to attend sessions during lunch breaks.

*“For me, I wouldn’t want to give up any of my time on any of my breaks or lunches to do anything outside what I’m already doing on my lunch or breaks.”* (AG13 FG)

Finally, agent’s engagement in the intervention and in particular, the education and training sessions appeared to raise their awareness of sitting, PA and the impact on health.

*“*[Engagement in the intervention] *pointed out to more myself and you as well (P10) and I’m expecting I presume whoever else is doing it, that how unhealthy were being just sitting, just sitting and eating and drinking, because you do that a lot because you’re sat at a desk,* […] *we do need to move and improve things for ourselves.”* (AG11 FG)

## Discussion

This mixed-methods study is the first to explore the acceptability and feasibility of a multi-component SB and PA intervention and associated evaluation, in the contact centre setting. The recruitment strategy in the present study needs refining to promote team leader interest, and avoid organisational procedures that prevent agents from engaging in recruitment sessions. While call agents perceived the data collection procedures feasible, strategies to increase adherence to pre-data collection fasting requirements are needed. Regarding the intervention components, education and training sessions, height-adjustable workstations and weekly emails respectively, were perceived most effective at supporting call agents to sit less and move more at work. The findings provide original evidence to the limited literature on PA and SB interventions in contact centres, and in accordance with guidance for intervention development [25], offer significant logistical and pragmatic considerations for future interventions in this setting.

Team leaders are perceived as pivotal in changing call agent perceptions of workplace PA and SB [18] and are frequently utilised in workplace interventions [61, 62]. Accordingly, to provide call agents in the present study with interpersonal support from their team leader, all team leaders were invited to participate, with only call agents in the team of an interested and eligible team leader subsequently invited to participate. This recruitment strategy contributed to only 30% of team leaders and 6% of call agents on the target office floor participating. Low team leader recruitment was attributed in part to the timing of recruitment, high workload, and a failure to engage team leader managers during recruitment. Thus, the pool of agents to recruit from was limited, with the agent recruitment rate below average compared to office-based trials (33%) [63]. Future similar trials are advised to recruit at the call agent level, or engage wider stakeholders to promote team leader buy-in, which appears consistent with employee perceptions from a previous workplace intervention [64]. In addition, implementing a compulsory team leader component may optimise call agent recruitment and promote greater consistency in intervention support given to agents by team leaders. To enable this, future trials are recommended to establish a clear overview of the organisational staffing structure and identify key stakeholders to engage with during a trials planning phase.

Call agent recruitment was further impacted by the exclusion of interested participants with a known cardiovascular or metabolic condition. This eligibility criterion is widely adopted in workplace interventions [15, 16, 65], however a review suggests that at risk populations can achieve greater glycaemic benefits following frequent breaks to sitting and light PA, compared to healthy individuals [66]. Further, the principle of proportional universality supports targeting the most at risk populations in order to yield the greatest proportional health benefits [67]. This poses an important consideration for eligibility criteria in trials to prevent the onset and treatment of chronic conditions. To that end, recruiting ‘healthy’ individuals without pre-existing cardiometabolic conditions may limit the apparent effectiveness of interventions on such health indicators. It may also limit the generalisability of the findings across contact centre call agents who have an elevated cardiometabolic risk compared to other occupational groups [7].

A red alert event in a contact centre results in the immediate removal of non-essential offline time for call agents. Red alert events are unique to contact centres compared to traditional offices, and in the present study, affected the research team’s ability to engage with call agents during recruitment drop-in and education and training sessions. Consequently, some agents’ exposure to the intervention was reduced, which could reduce intervention efficacy [25]. Red alert also occurred during data collection, which made it challenging to collect data in agents. Senior contact centre staff have identified that evidencing the impact of a PA or SB intervention is crucial if organisations are to adopt and implement the intervention [18], which is consistent with findings in a recent review [68]. Accordingly, researchers must be aware of red alert events in this setting, and work with contact centres to ensure offline time for call agents to engage in study procedures is protected.

Call agent attrition (48%) was largely due to job role changes and absence, with the attrition rate higher than a previous contact centre trial [8]. The average annual attrition in contact centres is 21%, with attrition often higher in the first 90 days of employment [69]. The high attrition rates observed in this sector and present study will make it challenging to evaluate long-term changes in behaviour and health, wellbeing, and productivity indicators, and this must be considered when planning sample sizes for future trials [30]. Agents generally perceived the 1-h data collection sessions as acceptable and feasible. Missing data was most prevalent for the 7-day activity monitoring, and blood pressure and blood sampling, with the latter due to participants forgetting to fast. Adherence to fasting requirements is essential for evaluating changes to fasting glucose, cholesterol and triglycerides, and the proposed strategy of text message reminders may reduce missing cardiometabolic data in future trials. Importantly however, the majority of agents felt comfortable with the data collection procedures employed.

Call agents perceived the education and training sessions, weekly emails and height-adjustable workstations as the most effective intervention components. The education sessions and weekly emails appeared to increase agents’ awareness of their PA and SB levels, and the workstations were perceived as a key enabler for reducing and breaking up sitting time. Similar to a previous trial [23], call agents found it easy to transition between seated and standing work with the workstation, with no adverse effects on productivity reported. Adopting a multi-level, multi-component approach appears promising for interventions in this setting and supports an ecological approach to real world intervention design [32].

Consistent with previous research, agents citied various health and work-related benefits to reduced sitting at work, including reduced musculoskeletal symptoms [70], improved health awareness [64] and reduced fatigue [12]. Novel benefits perceived by agents included improved optical health, and improved tone of voice, confidence and assertiveness during customer calls while standing compared sitting. Several agents felt this perceived confidence had a positive impact on their call control, and team leaders perceived their agents as more engaged and empowered when standing on calls, with suggestions of improved productivity. This perceived productivity finding is supported by objective data from a previous contact centre trial [22] and the collective findings suggest that height-adjustable workstations may be effective for reducing sitting time and increasing standing time in contact centres, while maintaining or improving productivity. Future trials should investigate changes in objectively measured productivity, PA and SB outcomes in call agents to support or refute this currently limited evidence, and inform the business case for contact centre interventions.

The observed perceived benefits support a preventative approach to implementing ergonomic aids within contact centres to optimise employee health and productivity. This is in contrast to current occupational and ergonomic policy that requires agents to have a pre-existing medical or musculoskeletal condition in order to receive adapted chairs or height-adjustable workstations [35]. Consistent with a recent review therefore [68], contact centre managers may benefit from greater education on the risks of high daily sitting to call agent’s cardiometabolic [60, 71] and musculoskeletal health [12], and the benefits of substituting sitting time with periods of standing and light PA [34]. Changing occupational policies and job roles to acknowledge PA and SB, and, providing support for agents and team leaders to implement strategies into daily working practices, could reflect this hazard accordingly and promote a shift away from sedentary working practices for a significant proportion of the adult working population [17].

Dealing with challenging customer calls was reported by agents as a key prompt to work in a standing position. To the authors’ knowledge, this original finding is unique to the contact centre setting, and contradicts observations in other desk-based workers who, with access to a height-adjustable workstation, reverted back to seated postures to conduct challenging or complex tasks [40]. This suggests that future contact centre trials can target the high volume of daily phone calls, especially challenging calls, as cues for agents to break up their sitting time. Interestingly, a high proportion of calls in this setting are complaints based, which exposes agents to frequent customer incivility that is reported to negatively influence wellbeing [71]. Standing on calls in the present study was perceived to increase agents’ confidence and assertiveness, and supports a recent trial that reported sitting reduction as a gateway to stress relief [72]. Accordingly, the promotion of standing-based work in contact centres may not only reduce sitting time, but support and protect call agents’ wellbeing, with further research required on this topic.

Seeing agents use a height-adjustable workstation in the standing position was a prominent trigger for agents to work in a standing position. Equally, low participant numbers meant that agents were often situated in teams of mainly desk-based agents, and similar to findings in traditional office workers [73, 74] and call agents [75], social pressure to conform to seated work appeared to negatively influence agent’s motivation to use the height-adjustable workstation in the standing position. Refining the recruitment strategy to increase agent participation and locate participants more proximally to one another appears important for increasing interpersonal support to use height-adjustable workstations in the standing position [33].

Consistent with previous research [76], agents identified that ownership of personal space, time to change between desks, and specialist equipment needs were barriers to using a height-adjustable workstation on a hot desk. Researchers and practitioners are therefore advised to provide contact centre call agents with individual workstations, as supported by previous research [33]. Height-adjustable workstations are however expensive, and cost is a barrier to employers investing in such equipment [19, 77]. Accordingly, future research should determine the cost-effectiveness of workplace trials that include the provision of individual height-adjustable workstations [78].

The ‘move more’ intervention aim appeared to lack consistent implementation in this study. Similar to findings from a workplace SB intervention [77], the movement champion in the present study was perceived to have low engagement with agents and focus on encouraging agents to sit less rather than move more. Further, reliance was placed on team leaders to implement standing or active meetings, and prompt agents to take active breaks. Replacing sitting time with standing may not be enough to elicit desired cardiometabolic adaptations in healthy individuals [79], and strategies to increase PA, in addition to SB reduction, are encouraged [66, 80]. To date though, effective and sustainable strategies for increasing workplace PA appear unknown [81]. Given call agents have low autonomy over their working practices and few opportunities to accrue incidental PA at work, future trials should explore the acceptability of refining or introducing organisational policies that may facilitate PA at work, such as frequent or longer breaks and greater task variation, alongside greater support and education for agents to be active during break times.

### Strengths and limitations

This is the first study to use mixed-methods to explore the acceptability and feasibility of an informed, multi-level, multi-component intervention, underpinned by behaviour change theory, in the unique and challenging contact centre setting [33]. The study adopted a pragmatic approach to implementing tailored intervention components to a real word setting, as guided by the Medical Research Council framework [24]. The process evaluation and engagement of multiple stakeholders to explore the acceptability and feasibility of the recruitment strategy, data collection procedures and intervention components has provided original knowledge to refine and justify the current intervention and improve its likely effectiveness and sustainability, which will be investigated in a future trial [24, 25]. One limitation of the study is the recruitment of a single contact centre who expressed an interest in the research. This introduces a potential bias towards the perceived acceptability and feasibility of the intervention components and methodology used in the present trial. Furthermore findings are limited by a small sample of call agents. Future trials should refine the recruitment processes as discussed, to optimise agent engagement and explore the feasibility of randomisation to a control group. Future trials exploring this can report on completion and attrition rates across treatment arms. Similar to previous trials [40], the study was conducted over 8-weeks, with longer term follow ups able to explore the sustainability and effectiveness of interventions [30]. Longer-term trials should consider the high attrition rate and transient workforce in contact centres compared to traditional office settings [69].

## Conclusions

This study has identified unique, pragmatic considerations for conducting a multi-level, multi-component PA and SB intervention and associated evaluation in highly sedentary call agents in the challenging contact centre setting. The intervention was perceived positively, with call agents and team leaders describing numerous perceived positive effects on behavioural, health and work-related outcomes. The findings provide evidence to refine the recruitment strategy to optimise agent engagement, enhance compliance to data collection requirements, and enhance the likely effectiveness and sustainability of the intervention components. Developing this complex intervention in an iterative manner, in accordance with frameworks for intervention development, has provided valuable considerations for tailoring future interventions to the contact centre setting, and the findings will be used by the current authors to refine and justify the design of a subsequent larger trial.

## Additional file

Additional file 1: **Table S1.** Participating call agents’ perceptions of the feasibility of data collection, perceptions of the height-adjustable workstations, and, willingness to continue to receive each intervention component. This table reports data based on a five point Likert scale ranging from strongly agree to strongly disagree. (DOCX 17 kb)

## Abbreviations

PA: Physical Activity
SB: Sedentary Behaviour
