# Supplementary material for: A multi-component intervention to sit less and move more in a contact centre setting: a feasibility study
Source: BMC Public Health. 2019 Mar 12;19:292. doi: 10.1186/s12889-019-6615-6 (PMC6416901; doi:10.1186/s12889-019-6615-6)
Supplement: Supplementary file 1 — Table S1. Participating call agents’ perceptions of the feasibility of data collection, perceptions of the height-adjustable workstations, and, willingness to continue to receive each intervention component. This table reports data based on a five point Likert scale ranging from strongly agree to strongly disagree. (DOCX 17 kb) [file 12889_2019_6615_MOESM1_ESM.docx]

| **Supplementary Table 1.**  **Participating call agents’ perceptions of the feasibility of data collection, perceptions of the height-adjustable workstations, and, willingness to continue to receive each intervention component.** | | | | | |
| --- | --- | --- | --- | --- | --- |
|  | **1** | **2** | **3** | **4** | **5** |
| *Feasibility of data collection* |  |  |  |  |  |
| It was feasible for me to provide my body stature measurements (height, weight, waist circumference, hip circumference). | 100% | - | - | - | - |
| It was feasible for me to have my blood pressure taken. | 100% | - | - | - | - |
| It was feasible for me to complete the surveys. | 100% | - | - | - | - |
| It was feasible for me to come into work in a fasted state. | 73% | 18% | 9% | - | - |
| It was feasible for me to provide a 15ml blood sample. | 91% | - | - | 9% | - |
| It was feasible for me to wear an accelerometer for 7 days. | 82% | 9% | - | 9% | - |
| I felt supported by my organisation to complete the assessment protocol within work hours. | 91% | 9% | - | - | - |
|  |  |  |  |  |  |
| *Perceptions of the height-adjustable workstations* |  |  |  |  |  |
| The height adjustable workstation is easy to use. | 90% | 10% | - | - | - |
| I felt comfortable using the height adjustable workstation in the presence of others at my work. | 90% | - | 90% | - | - |
| My work-related productivity decreased while using the height adjustable workstation. | - | - | 10% | 90% | - |
| The quality of my work decreased while using the height adjustable workstation. | - | - | - | 10% | 90% |
| I was more tired on days I used the height adjustable workstation. | 22% | 11% | 22% | 44% | - |
| I had more musculoskeletal troubles on days I used the height adjustable workstation, for example back, muscle or joint pain, height adjustable workstation. | 22% | 11% | - | 67% | - |
| I would welcome further advice and guidance for using the height adjustable workstations to optimise health gains. | 51% | 20% | 20% | - | 9% |
|  |  |  |  |  |  |
| *Willingness to continue to receive each intervention component* |  |  |  |  |  |
| I would be willing to continue having access to the height adjustable workstation. | 91% | - | - | 9% | - |
| If I were offered a height adjustable workstation for my own desk, from my employer, I would take up the offer. | 91% | 9% | - | - | - |
| I would be willing for the movement champion to continue in their role. | 82% | 18% | - | - | - |
| I would be willing to receive weekly emails from the team leader. | 73% | 27% | - | - | - |
| I would be willing to keep physical activity and sedentary behaviour as a discussion point in the weekly team meetings. | 50% | 33% | - | 17% | - |
| I would be willing to continue my walking one-to-one’s with my team leader. | 50% | 13% | 25% | 13% | - |
| I would be willing to attend further education and training sessions on health and physical activity. | 91% | 9% | - | - | - |
| Note: Totals may not equal 100% due to statistical rounding.  1 = strongly agree, 2 = agree, 3 = neutral, 4 = disagree, 5 = strongly disagree. | | | | | |
